# Clinical Impact and Significant Technical Points of Transarterial Chemoembolization (TACE) Using the Smaller Drug-Eluting Bead M1 (DC Bead M1™) for Hepatocellular Carcinoma: A Case Series

**DOI:** 10.7759/cureus.74415

**Published:** 2024-11-25

**Authors:** Yusuke Kawamura, Norio Akuta, Shigeki Yamamoto, Yasuka Eriksson, Tetsuya Hosaka, Satoshi Saitoh, Hitomi Sezaki, Fumitaka Suzuki, Kenji Ikeda, Hiromitsu Kumada

**Affiliations:** 1 Hepatology, Toranomon Hospital, Tokyo, JPN; 2 Okinaka Memorial Institute for Medical Research, Toranomon Hospital, Tokyo, JPN

**Keywords:** advanced hepatocellular carcinoma, dc bead m1, deb-tace, dem1-tace, drug-eluting bead, epirubicin, systemic therapy

## Abstract

Background: This case series evaluated the clinical impact and significant technical points of transarterial chemoembolization (TACE) for hepatocellular carcinoma (HCC) using the smaller drug-eluting bead (DEB) M1 (DC Bead M1^TM^; 70-150 µm).

Methods: We evaluated 12 patients and 14 HCC nodules treated with DEB-TACE using the DC Bead M1^TM^ (named DEM1-TACE). In addition to evaluating the early treatment efficacy for each treated node after DEM1-TACE, the study also used interventional radiology (IVR)- computed tomography (CT) to focus on the presence or absence of retention of the homogeneous contrast medium in target nodules after DEM1-TACE as a predictor of a good treatment response.

Results: Nine HCC nodules (64%) showed a complete response by modified Response Evaluation Criteria in Solid Tumors (mRECIST), while two nodules (14%) had a partial response. Finally, 11 nodules (79%) showed an objective response (OR). Moreover, IVR-CT showed target nodules with retention of homogeneous contrast medium after DEM1-TACE, eight of nine (89%) nodules achieved a complete response and nine of nine (100%) nodules showed an OR, resulting in an objective response rate (ORR) of 100%. In contrast, in nodules without retention of homogeneous contrast medium in treated target nodules after DEM1-TACE, two of five (40%) nodules showed OR, resulting in an ORR of 40%. The ORR was significantly higher in the group with retention of homogeneous contrast medium after DEM1-TACE (*P* = 0.028).

Conclusions: DEM1-TACE had good treatment responses in patients with HCC. Evaluating retention of homogeneous contrast medium after DEM1-TACE using IVR-CT is one of the main predictors of treatment success.

## Introduction

Transarterial chemoembolization (TACE) is currently recommended for the treatment of intermediate-stage hepatocellular carcinoma (HCC) in the Barcelona Clinic Liver Cancer (BCLC) system [1]. However, in the current treatment strategy for HCC, TACE is often performed in combination with systemic therapy in order to control intrahepatic lesions in the intermediate stage (stage B) and advanced stage of BCLC (stage C) [2-6]. In addition, TACE using drug-eluting beads (DEBs) (DEB-TACE) is attracting attention as a useful combination of systemic therapy and TACE [3,4,7], with its synergistic effect with immunotherapy also expected [8].

In general, smaller diameter beads are recommended for TACE of HCC [9], and until now, small beads (100-300 µm) have been used mainly in Japan. However, since June 2024, smaller beads (DC Bead M1^TM^; 70-150 µm) have been available in Japan, and it is expected in the future that these beads will be the main size used in DEB-TACE for HCC.

Current evidence from the PRECISION V study, a randomized controlled trial, demonstrated that TACE using the DC Bead^TM^ had a higher response rate than conventional TACE using lipiodol and a variety of embolic substances, although statistical difference was not obtained between the two treatment arms [10]. On the other hand, the inferiority of DEB-TACE compared to cTACE has also been reported recently in relatively small tumors and also low numbers of tumors [11]. We speculate that these contradictory results are due not only to tumor factors such as the size and number of HCCs but also to a range of issues such as the essential nature of the DEB-TACE procedure and differences in the appropriate size of the DC bead for each tumor.

A smaller DC Bead M1^TM^ is now available and is expected to improve the therapeutic effect of DEB-TACE. On the other hand, when considering the history of DEB-TACE, there is a need for early validation of a treatment method that potentially has a higher response rate and also to develop a method for predicting the treatment response. The current study was therefore conducted to investigate and determine these two clinical parameters.

## Materials and methods

Study population

Between May 2024 and September 2024, 12 patients with HCC and a total of 14 nodules received DEB-TACE using DC Bead M1^TM^ (named DEM1-TACE). All these patients were included in this case series study. For inclusion in the study, the patients were required to meet the following conditions: (1) the presence of a typical HCC diagnosed by either dynamic-computed tomography (CT) or magnetic resonance imaging (MRI) before the TACE procedure; (2) all target nodules had no history of receiving DEB-TACE before DEM1-TACE; (3) all target nodules received selective TACE at the subsegmental branch or deeper; (4) A maximum target tumor number of two; and 5) an observation period of ≥4 weeks. 

All the procedures were carried out in accordance with the ethical standards of the responsible committees for human experimentation (institutional and national) and the criteria of the 1975 Helsinki Declaration. The study was approved by the Institutional Review Board of our hospital (protocol number, 1438-H/B).

Diagnosis of HCC

Analysis of dynamic CT or MRI images was used to diagnose HCC. A nodule was diagnosed as HCC if dynamic imaging showed hyperattenuation in the arterial phase and washout in the portal or delayed phase.

Assessment of tumor localizations

The usefulness of zoning of tumor location (central or peripheral tumor) for estimating the treatment success of TACE was reported recently [12]. In this zoning criteria, the central tumor is defined as the lesion located even partially within 1 cm of the main trunk or first branch of the portal vein, while the peripheral tumor is defined as the lesion located, even in part, more than 1 cm away from the main trunk or the first branch of the portal vein. The current study therefore evaluated the tumor zoning.

Preparation and treatment protocol for DEM1-TACE

Epirubicin (Farmorubicin^TM^, Pfizer Japan, Tokyo, Japan) is a highly water-soluble, antitumor agent used for the treatment of HCC. A total of 50 mg of epirubicin was reconstituted in 2 mL of water for injection (Otsuka Pharmaceutical, Tokyo, Japan). Six mL of phosphate buffer saline was removed from a vial containing DC Bead M1^TM^ (Boston Scientific, Marlborough, MA, USA), followed by the addition of 2 mL of reconstituted epirubicin solution. The DC Bead M1^TM^ and epirubicin solution was then agitated for 30 seconds using a Vortex mixer (Vortex-Genie 2, Scientific Industries, Philadelphia, USA). After five minutes standing from the finish of agitation, the total volume of DC Bead M1^TM^ decreased from 2 to 1.4 mL (i.e., almost 30% decrease). The final volume eventually decreased to 1 mL due to adhesion of the solution to the vial wall and hub. A 19 mL aliquot of contrast medium was then loaded into 1 mL of the epirubicin-loaded DC Bead M1^TM^ solution to make a total volume of 20 mL (dilution ratio 20x; diluted solution A). Finally, we created a diluted solution at a dilution factor of 200x.

**Figure 1:**
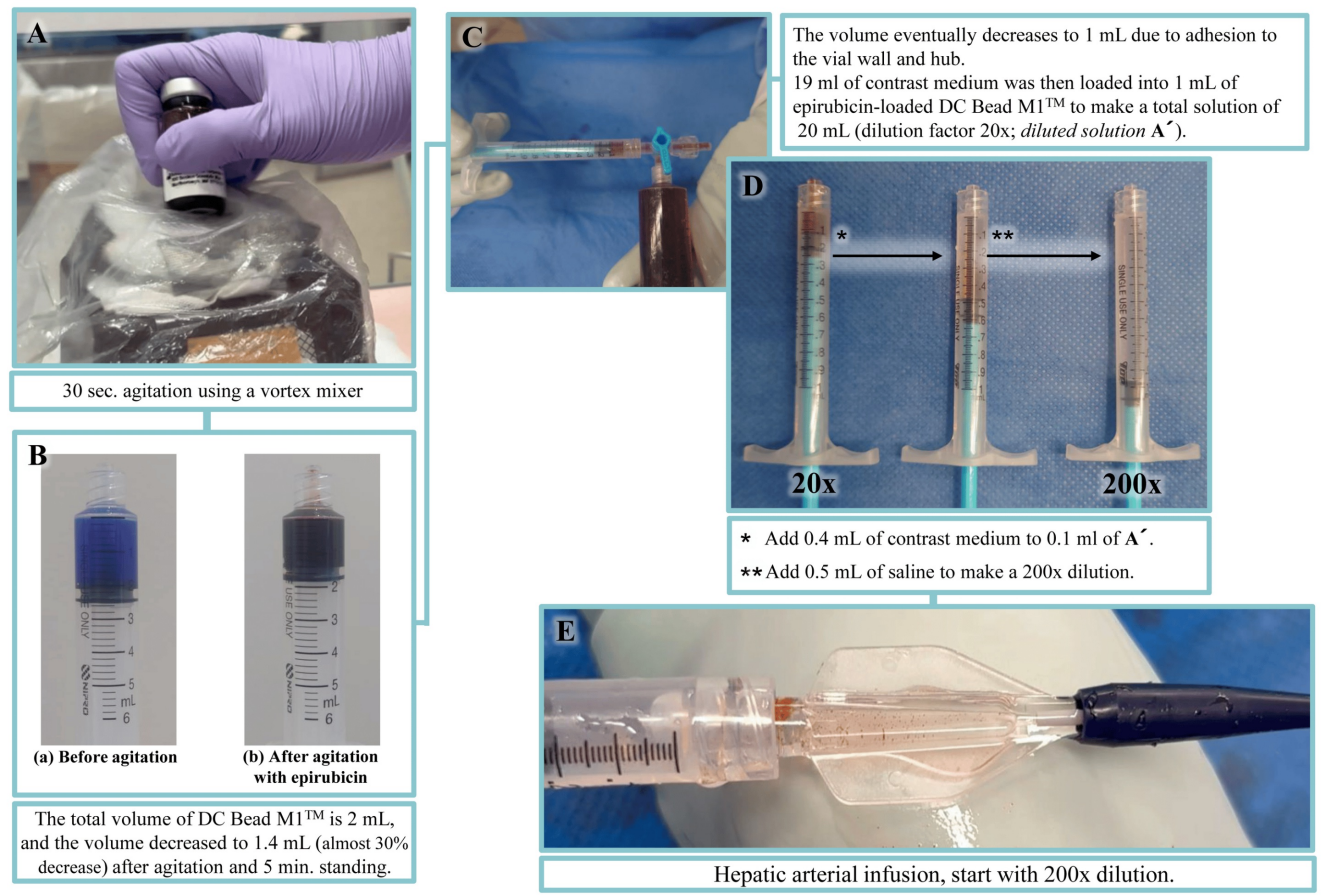
Preparation and treatment protocol for DEM1-TACE DEM1-TACE: drug-eluting bead (DEB)-TACE using DC Bead M1^TM^ (A) Agitation using a Vortex mixer, (B) changes in the total volume of DC Bead M1^TM^ after agitation, (C) dilution step 1, (D) dilution step 2, (E) hepatic arterial infusion

The protocol for DEM1-TACE was to start with a dilution rate of 200x, and if the reduction in arterial blood flow was insufficient, the dilution factor was reduced (usually from 200x to 100x) (Figure 1).

To maintain visibility during infusion and to ensure safe treatment, the dilution factor of the contrast agent was maintained below 50% during treatment. The endpoint of treatment was the disappearance of tumor staining, with the residual tumor vessels allowed to remain after embolization.

After DC Bead M1^TM^ infusion, soft embolization with 1 mm gelatin particles (Gelpart^®^; Nippon Kayaku, Tokyo, Japan) was added to temporarily stop arterial blood flow. In cases when the vascular lake phenomenon (VLP) appeared, embolization using gelatin particles was added until the VLP disappeared. The entry site of TACE (transfemoral or transradial) was decided based on our previous report [13].

Image acquisition protocol during the DEM1-TACE procedure

All patients in the study underwent the DEM1-TACE procedure using an angiographic device (Artis zee i TA; Siemens Healthineers, Erlangen, Germany) and an interventional radiology (IVR)-CT device (SOMATOM Definition AS+ 128; Siemens Healthineers, Erlangen, Germany). The vascular lake during the DEM1-TACE procedure was evaluated by digital subtraction angiography (DSA) imaging. After the DEM1-TACE procedure, all the patients received an IVR-CT examination to evaluate the degree of contrast medium retention in each target nodule.

Typical types of contrast medium retention patterns and the treatment effect evaluated by IVR-CT in target nodules after DEM1-TACE

**Figure 2:**
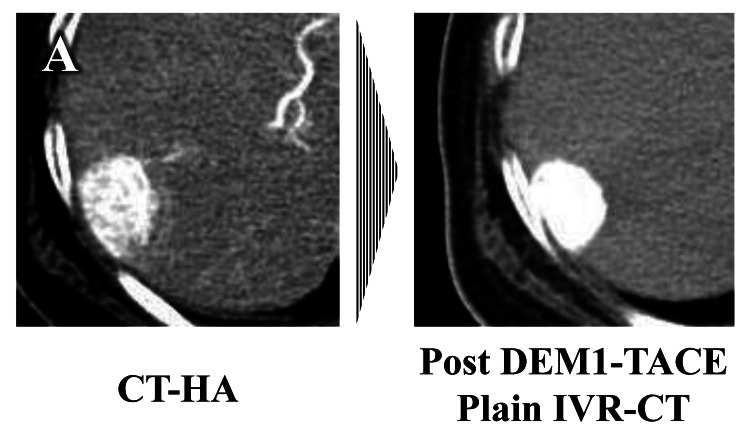
Typical pattern of homogeneous contrast medium retention evaluated by IVR-CT in target nodules after DEM1-TACE CT-HA: computed tomography-during hepatic arteriography; DEM1-TACE: drug-eluting bead (DEB)-TACE using DC Bead M1^TM^; IVR-CT: interventional radiology-computed tomography (A) Homogeneous contrast medium retention appeared in IVR-CT after DEM1-TACE

**Figure 3:**
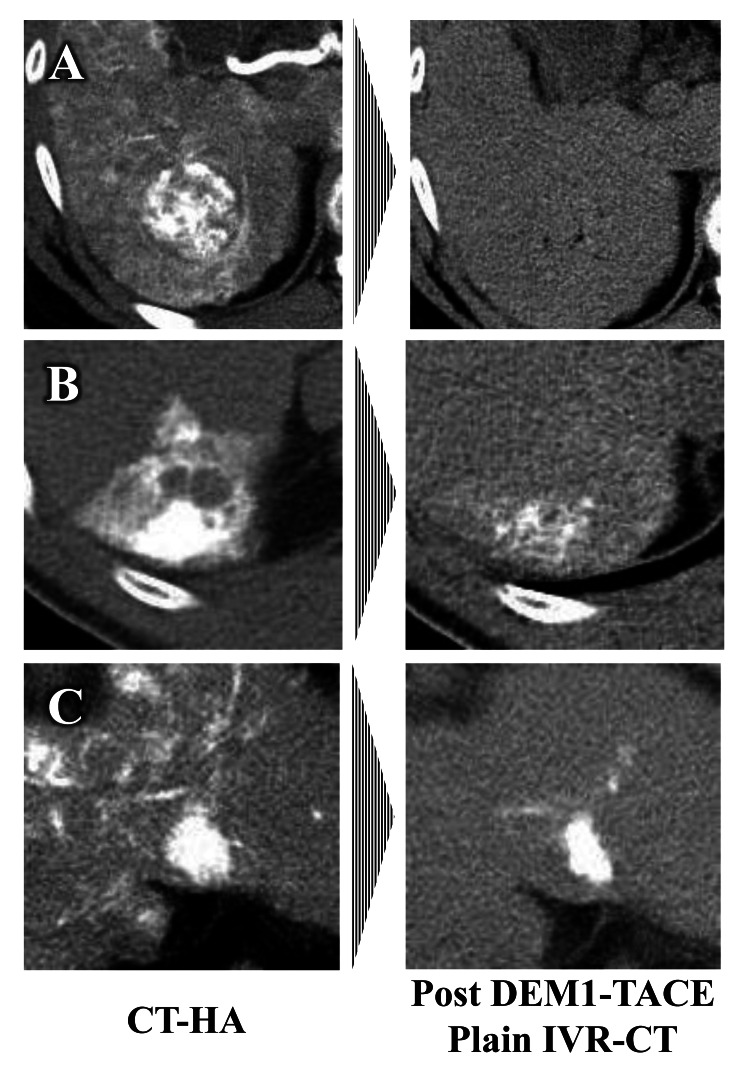
Typical patterns of nonhomogeneous contrast medium retention evaluated by IVR-CT in target nodules after DEM1-TACE CT-HA: computed tomography-during hepatic arteriography; DEM1-TACE: drug-eluting bead (DEB)-TACE using DC Bead M1^TM^; IVR-CT: interventional radiology-computed tomography Insufficient contrast medium retention caused by (A) larger tumor vessel diameter, (B) histological nature of the tumor, and (C) insufficient embolization

The typical types of contrast medium retention patterns evaluated by IVR-CT in target nodules were classified into two patterns. Pattern 1 represented homogeneous retention of contrast medium in the target nodule (Figure 2), and pattern 2 represented the absence of homogeneous contrast medium retention. Pattern 2 included heterogeneous contrast medium retention in target nodules and insufficient contrast medium retention (Figure 3).

These contrast medium retention patterns were assessed independently by the following hepatologists YK, SY, and EY. Discrepancies between these three examiners were resolved by consensus review including an additional reviewer KI.

Assessment of therapeutic effects and follow-up

The early treatment efficacy in each nodule was evaluated by dynamic CT approximately one month after DEM1-TACE in accordance with the modified Response Evaluation Criteria in Solid Tumors (mRECIST) guidelines [14]. Imaging studies were carried out every one to two months after the initial evaluation.

Statistical analysis

The statistical analyses were performed using IBM SPSS Statistics for Windows, Version 30 (Released 2021; IBM Corp., Armonk, New York, United States). The data were expressed as the median and range. Differences in background features between each parameter and treatment efficacy were analyzed using Fisher’s exact test and Mann-Whitney U test. The significance of changes in aspartate aminotransferase (AST) levels, modified albumin-bilirubin (mALBI) score, and renal function tests before and after DEM1-TACE was evaluated using the Wilcoxon signed-rank test. A p-value of <0.05 was considered to denote a statistically significant difference.

## Results

Overview

Table 1 summarizes the baseline characteristics of the patients and target nodules before DEM1-TACE. Regarding the BCLC stage, 11 patients showed very early or early stage (stages 0 and A) (92%), with no patient having advanced stage (BCLC stage C). Nine patients were classified as Child-Pugh class A, while eight patients showed good liver function classified as mALBI grades 1 and 2a (67%). Four patients selected the transradial approach (33%) for TACE. For nodule tumor characteristics, the median tumor diameter was 21.0 mm, with two nodules (14%) localized in the central zone.

**Table 1:** Clinical profiles and laboratory data of HCC patients treated with DEM1-TACE AFP: alpha-fetoprotein; AST: aspartate aminotransferase; BCLC: Barcelona Clinic Liver Cancer; DCP: des-g-carboxyprothrombin; DEM1-TACE: drug-eluting bead-TACE using DC Bead M1^TM^; HBV: hepatitis B virus; HCC: hepatocellular carcinoma; HCV: hepatitis C virus; IU: international units; mALBI: modified albumin-bilirubin; NonB, NonC: neither HBV nor HCV infection present; TACE: transarterial chemoembolization †Data expressed as median (range) The ratios are rounded off to the first decimal place, and therefore, the total will not necessarily be 100

Patient characteristics and laboratory data (n = 12)		
Sex, n (%)	Males	9 (75%)
	Females	3 (25%)
Age group (in years)	<70	3 (25%)
	71-80	5 (42%)
	81-90	3 (25%)
	>90	1 (8%)
Etiology, n (%)	HCV	8 (67%)
	HBV	2 (17%)
	NonB, NonC	2 (17%)
Other laboratory data	Platelet count, ×10^3^/mL (range)^†^	156 (61-199)
	Albumin, g/dL (range)^† ^	3.7 (2.9-4.0)
	Total bilirubin, mg/dL (range)^†^	0.7 (0.4-1.8)
	Prothrombin activity, % (range)^†^	79.2 (51.8-98.9)
	AST, IU/L (range)^†^	25 (12-77)
Tumor marker	AFP, µg/L (range)^†^	8.9 (1.9-313.5)
	DCP, AU/L (range)^†^	25.0 (7.0-132.0)
Child-Pugh class, n (%)	A	9 (75%)
	B	3 (25%)
	C	0 (0%)
BCLC stage, n (%)	0	5 (42%)
	A	6 (50%)
	B	1 (8%)
	C	0 (0%)
mALBI grade, n (%)	1	1 (8)%
	2a	7 (58)%
	2b	4 (33)%
	3	0 (0)%
Tumor characteristics per procedure (n = 14)		
Features	Largest tumor diameter, mm (range)^†^	21.0 (6.0-42.0)
	Macrovascular invasion, n (%)	0 (0%)
Locations	Central zone, n (%)	2 (14%)
	Peripheral zone, n (%)	12 (86%)
Pretreatment dynamic study enhancement patterns	Homogeneous, n (%)	6 (43%)
	Heterogeneous, n (%)	8 (57%)

Early treatment effect of DEM1-TACE according to the retention of dense contrast medium in the target nodule after procedure

Table 2 shows the early treatment effect of DEM1-TACE in mRECIST.

**Table 2:** Early treatment efficacy of DEM1-TACE according to contrast medium retention in treated HCC CR: complete response; DEM1-TACE: drug-eluting bead (DEB)-TACE using DC Bead M1^TM^; HCC: hepatocellular carcinoma; mRECIST: modified Response Evaluation Criteria in Solid Tumors; OR: objective response; ORR: objective response rate; PD: progressive disease; PR: partial response; SD: stable disease; TACE: transarterial chemoembolization ^†^The ORR was significantly high in the presence of homogeneous contrast medium retention in treated HCC after DEM1-TACE (*P* = 0.028)

	Response evaluation using mRECIST (per nodule), n (%)			
Homogeneous contrast medium retention in treated HCC after DEM1-TACE	CR	PR	SD	PD
Presence	8 (89%)	1 (11%)	0 (0%)	0 (0%)
	OR (ORR)^†^		non-OR (non-ORR)^†^	
	9 (100%)		0 (0%)	
Absence	1 (20%)	1 (20%)	3 (60%)	0
	OR (ORR)^†^		non-OR (non-ORR)^†^	
	2 (40%)		3 (60%)	

**Figure 4:**
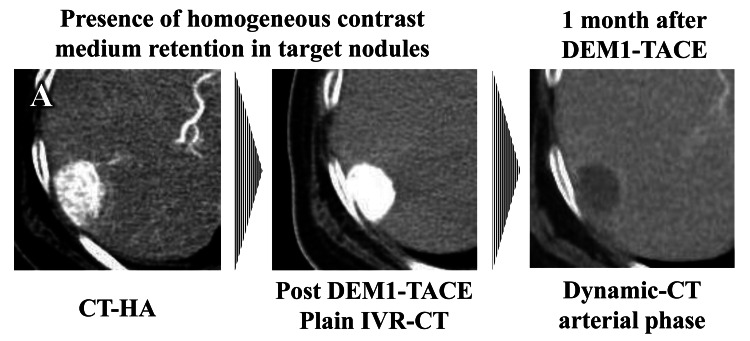
Typical treatment outcomes of homogeneous contrast medium retention patterns evaluated by IVR-CT in target nodules after DEM1-TACE CT-HA: computed tomography-during hepatic arteriography; DEM1-TACE: drug-eluting bead (DEB)-TACE using DC Bead M1^TM^; IVR-CT: interventional radiology-computed tomography; mRECIST: modified Response Evaluation Criteria in Solid Tumors (A) Target nodule showed complete response in mRECIST after DEM1-TACE

**Figure 5:**
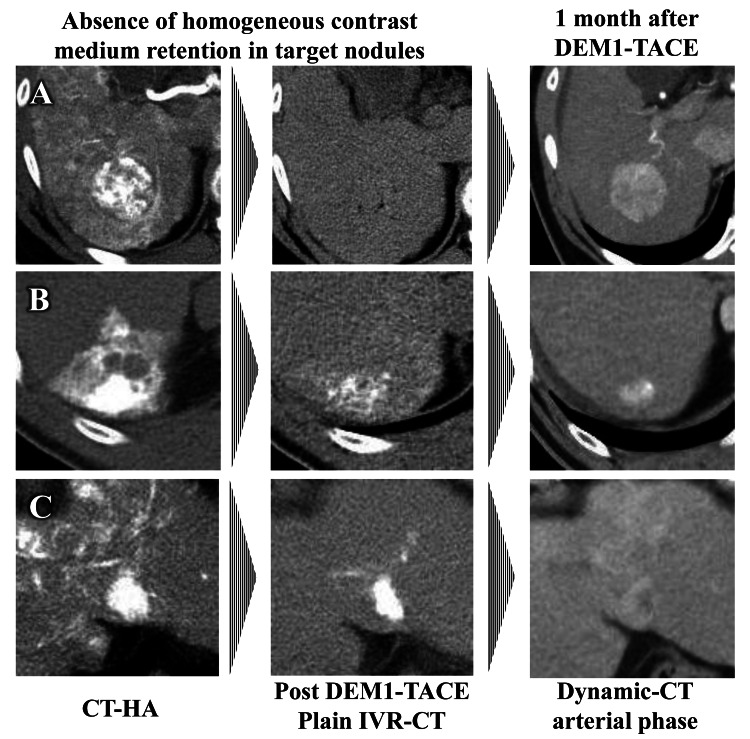
Typical treatment outcomes of nonhomogeneous contrast medium retention patterns evaluated by IVR-CT in target nodules after DEM1-TACE CT-HA: computed tomography-during hepatic arteriography; DEM1-TACE: drug-eluting bead (DEB)-TACE using DC Bead M1^TM^; IVR-CT: interventional radiology-computed tomography; mRECIST: modified Response Evaluation Criteria in Solid Tumors Insufficient treatment effect in mRECIST may caused by (A) larger tumor vessel diameter, (B) histological nature of the tumor, and (C) insufficient embolization

In patients with retention of homogeneous contrast medium in treated target nodules after DEM1-TACE (Figure 4), eight of the nine nodules achieved a complete response and nine of the nine nodules showed an objective response (OR), resulting in an objective response rate (ORR) of 100%. In contrast, in patients without retention of homogeneous contrast medium in treated target nodules after DEM1-TACE (Figure 5), the ORR was 40%. This resulted in significant differences in ORR between the presence or absence of contrast medium retention after DEM1-TACE (*P *= 0.028), with the final ORR of DEM1-TACE being 79%. In contrast, there were no significant differences in ORR between central and peripheral tumor locations (ORR: 100% and 75%, respectively; *P *= 1.000), and homogeneous and heterogeneous pretreatment dynamic study enhancement patterns (ORR: 100% and 63%, respectively; *P *= 0.209). There were also no significant differences in ORR between the presence or absence of VLP (ORR: 71% and 57%, respectively; *P *= 1.000).

This study's median volume of diluted solution A' used during the DEM1-TACE was 2 mL, with a minimum of 0.8 mL and a maximum of 4 mL.

Safety analysis of DEM1-TACE

**Figure 6:**
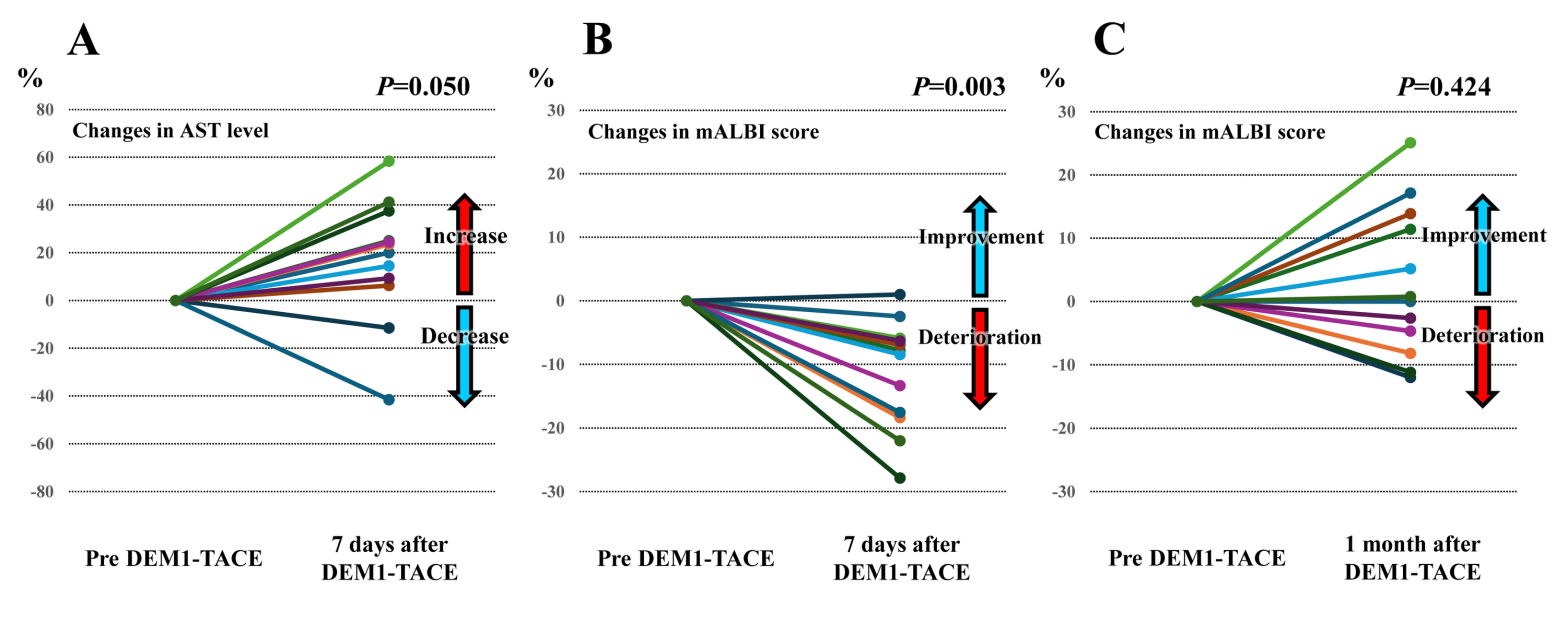
Changes in liver functional tests before and after DEM1-TACE DEM1-TACE: drug-eluting bead (DEB)-TACE using DC Bead M1^TM^; mALBI: modified albumin-bilirubin; AST: aspartate aminotransferase (A) Changes in AST level one week later, (B) changes in mALBI score one week later, and (C) changes in mALBI score one month later

**Figure 7:**
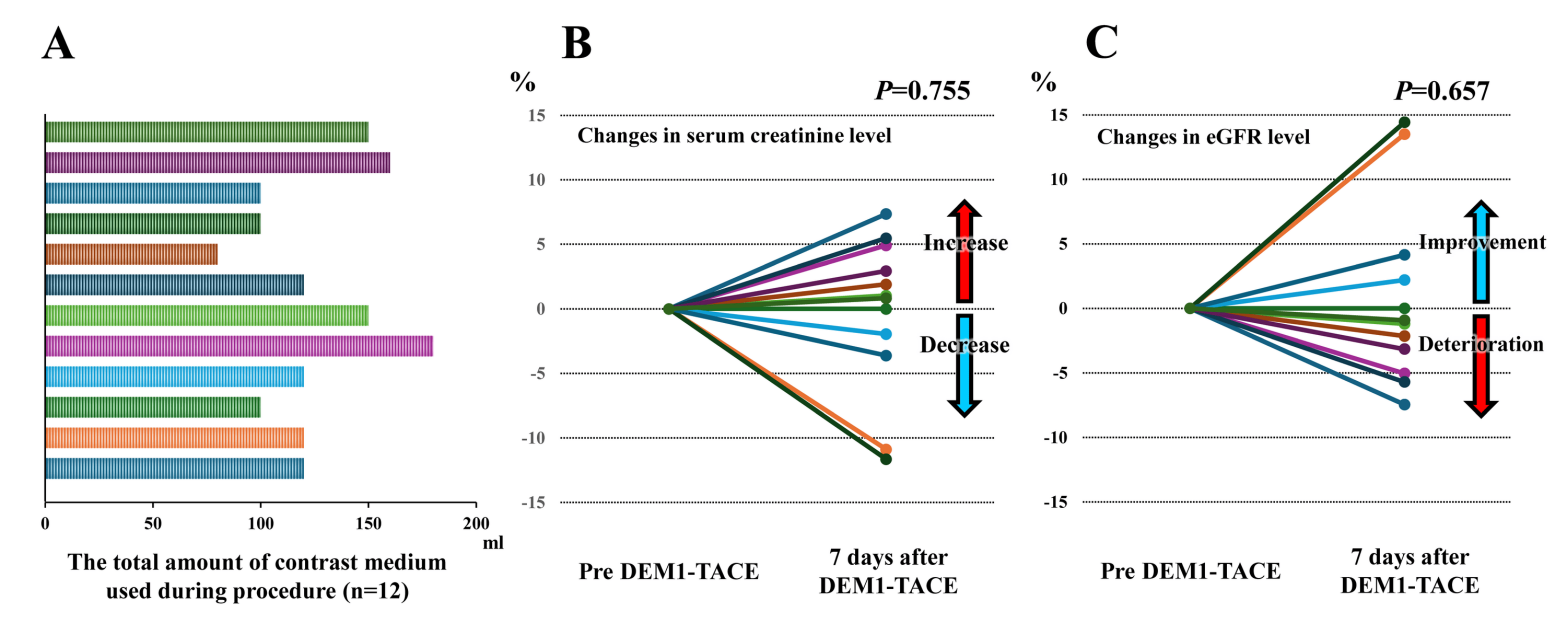
Changes in renal functional tests before and after DEM1-TACE and the total amount of contrast medium used during the procedure DEM1-TACE: drug-eluting bead (DEB)-TACE using DC Bead M1^TM^; eGFR: estimated glomerular filtration rate (A) The total amount of contrast medium used during the procedure, (B) changes in serum creatinine level one week later, and (C) changes in eGFR level one week later

Figure 6 shows the changes in AST level and mALBI score before and after DEM1-TACE. The AST level tends to increase after DEM1-TACE (*P *= 0.050), and there were significant changes in the mALBI score before and after DEM1-TACE (*P *= 0.003). However, the mALBI score improved after one month. These results indicate that the post-embolization syndrome was as mild as that achieved by conventional DEB-TACE, with no severe adverse effects being observed. Figure 7 shows the changes in renal functional tests before and after DEM1-TACE and the total amount of contrast medium used during the procedure. This study's median volume of contrast medium used during the procedure was 120 mL. There were no significant changes in serum creatinine and estimated glomerular filtration rate (eGFR) levels before and after DEM1-TACE (*P *= 0.755 and 0.657, respectively). In addition, there were no complications related to the procedure, including cerebral infarction.

## Discussion

The PRECISION V study, a randomized, controlled trial, demonstrated that TACE using DC Beads had a higher response rate than that of conventional TACE using lipiodol and varied embolic substances, although there was no statistical difference observed between the two treatment arms [10]. On the other hand, it has been reported recently that DEB-TACE is inferior to cTACE for treating relatively small tumors and also low numbers of tumors [11]. These divergences in the therapeutic efficacy of DEB-TACE may be reduced with the advent of the smaller diameter DC Bead M1^TM^.

The results of the current study showed encouraging higher treatment effects with the use of DEM1-TACE for HCC. However, we consider that there are several points that need attention to obtain a stable and high therapeutic effect with DEM1-TACE. Firstly, a low dilution rate of bead particles easily forms clusters due to the structure of the particles that often become stuck in vascular branches and embolize proximally, thereby reducing the direct therapeutic effect on HCC. Before the advent of DC Bead M1^TM^, the recommended bead size was 100-300 µm for DEB-TACE of HCC [9,15]. Moreover, product information shows that the smaller DC Bead (100-300 µm) contains a higher number of particles per vial than that of the larger DC Bead (300-500 µm) (200,000 beads vs. 38,000 beads, respectively). It is therefore recommended that a dilution rate of 100x or higher is required for the smaller bead for standard DEB-TACE of HCC [15]. The DC Bead M1^TM^ (70-150 µm) also contains more beads per vial (1.3 million beads based on product information). Therefore, the dilution rate is an extremely important factor when considering DEB-TACE, especially when using DC Bead M1^TM^. However, many clinical reports have not described this important point and treatment process. The current study therefore returned to the basics of DEB-TACE to examine dilution rates, treatment endpoints, and radiological characteristics that may predict the treatment efficacy of DC Bead M1^TM^.

In principle, in treatment using DC Bead M1^TM^ at a sufficiently high dilution rate (i.e., 200x in this study), the risk of cluster formation is expected to be relatively low because of the small particle size and their rapid distribution in the arterial bloodstream into the peripheral tumor vessels after hepatic arterial infusion. As a consequence, we expected that the effect of the treatment would be equalized among surgeons if the procedure was performed in a similar manner.

Secondly, the utility of evaluating contrast medium retention after DEM1-TACE using IVR-CT requires consideration. Until now, the treatment endpoint of DEB-TACE was recommended as follows: “injection should be continued until near stasis is observed in the artery directly feeding the tumor (i.e., the contrast column should clear within 2-5 heartbeats)” [16]. On the other hand, when determining treatment endpoints in actual clinical practice, there is an expectation for objective endpoint indicators that are more closely related to response rates. The results of this study showed that retention of homogeneous contrast medium in IVR-CT into target nodules was associated with a good early treatment effect. We consider that this phenomenon is a simple and objective indicator for demonstrating a favorable treatment endpoint. In contrast, we observed no significant differences in ORR between the presence or absence of VLP (*P* = 1.000). According to a previous report, the VLP is a useful predictor for estimating successful treatment effects in conventional DEB-TACE and occurs readily in larger tumors more than 30 mm [17]. The median tumor diameter in our study cohort was relatively small at almost 20 mm, with VLP observed in seven of 14 (50%) nodules and the ORR being five of seven (71%) in the VLP group and four of seven (57%) in the non-VLP group. It is possible that these differences may have affected the study results. In addition, we observed no significant differences in ORR between central and peripheral tumor locations (*P* = 1.000). In general, tumor locations are very important factors for acquiring a complete response. Moreover, in pretreatment dynamic study enhancement patterns, six of six (100%) nodules with homogeneous enhancement pattern achieved OR, and five of eight (63%) nodules with heterogeneous enhancement pattern achieved OR. There were no significant differences in ORR between homogeneous and heterogeneous enhancement patterns (*P* = 0.209). In general, dynamic study enhancement patterns are essential predictive factors for estimating tumor differentiation and macroscopic growth types [18]. However, our study was unable to evaluate these points because of the small number of cases.

Finally, we experienced an insufficient treatment effect in three of 14 (21%) nodules. At this stage, we identified three factors that may have caused insufficient treatment effects, the first being insufficient embolization area, the second the histological nature of the tumor, and the third tumor vessel diameter.

**Figure 8:**
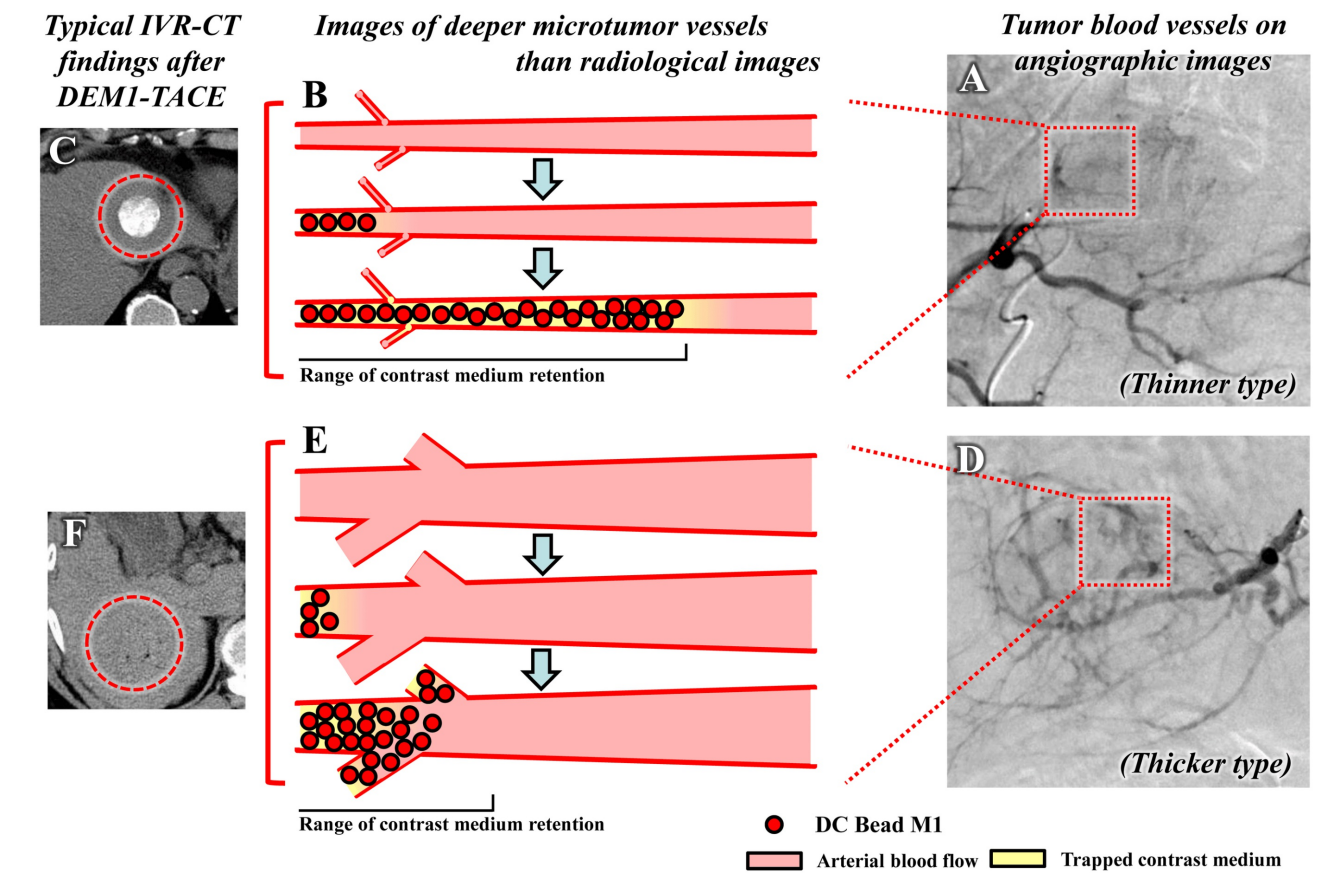
Estimated relationship between tumor blood vessels on angiographic images and contrast medium retention after DEM1-TACE DEM1-TACE: drug-eluting bead (DEB)-TACE using DC Bead M1^TM^; IVR-CT: interventional radiology-computed tomography (A) Thinner type of tumor blood vessels on angiographic images. (B) Sufficient range of contrast medium retention for acquiring homogeneous contrast medium retention after DEM1-TACE. (C) Homogeneous contrast medium retention appeared after DEM1-TACE in the target nodule (red-dotted circle). (D) Thicker type of tumor blood vessels on angiographic images. (E) Insufficient range of contrast medium retention for acquiring homogeneous contrast medium retention after DEM1-TACE. (F) Insufficient contrast medium retention observed after DEM1-TACE in the target nodule (red-dotted circle)

Regarding the tumor vessel factor, we speculate that the temporary loss of tumor staining in DSA may be due to successful deeper occlusion of tumor vessels near the tumor. However, longer distance occlusion of tumor vessels may be necessary to demonstrate retention of contrast medium in the target nodule viewed by IVR-CT immediately after embolization. In the analysis of the short axis of tumor blood vessels diameter on angiographic image, high attenuation of contrast medium on IVR-CT was easy to observe when the tumor diameter was smaller than 0.6 mm (i.e., thinner type). On the other hand, in this analysis, there was no high attenuation of contrast medium on IVR-CT in radiological tumor diameter greater than 1.9 mm (i.e., thicker type) (Figure 8).

The results of the analysis of tumor blood vessel diameter suggested that larger beads may be appropriate for thicker tumor blood vessels with a diameter greater than 1.0 mm. Therefore, evaluation of tumor blood vessel size during DSA imaging may assist in selecting the appropriate bead size for treatment. However, in this study, the median diameter of the target tumor was 21 mm, which is relatively small. Due in part to the relatively small tumor size and small number of cases, there was no significant difference in tumor size between the OR group (median tumor diameter 20 mm) and the non-OR group (median tumor diameter 26 mm) in this study (*P* = 0.180). Hence, we consider that more cases are needed to verify the appropriate bead size for a specific tumor blood vessel diameter.

In this study, we performed 30 seconds of vial shaking using a Vortex mixer in accordance with previous reports (Figure 1) [19]. However, based on recently published basic research, the most convenient and effective way for reducing the loading time of epirubicin into DC Bead M1^TM^ was “left at room temperature for five minutes” [20]. Therefore, we consider that the vial shaking phase using a Vortex mixer could be converted to “left at room temperature for five minutes” in future daily clinical practice.

In the era of systemic therapy for HCC, controlling intrahepatic targets by combining systemic therapy and locoregional treatment with less impact on liver function is attracting attention. DEM1-TACE is a useful treatment method that has less of an impact on liver function, but the results of this study are limited to small size and small numbers of nodules. On the other hand, it has been reported that transarterial chemotherapy using cisplatin is a valuable treatment option for HCC with a high tumor burden [21,22]. Further research is required to determine how to combine the use of these catheter treatments with systemic therapy differently depending on various tumor and liver functional conditions, in the future.

Finally, as the results of this study showed that DEM1-TACE had a high therapeutic effect, we are now performing further research to evaluate the utility of the combined use of systemic therapy and DEM1-TACE. The combined use of lenvatinib is named LEM1-TACE, while atezolizumab plus bevacizumab is named AM1-TACE. 

This study had several limitations that included its retrospective nature, performance at only a single center, and the relatively small number of patients. Hence, there are limitations in the statistical considerations. In addition, the median tumor size was relatively small, and there was only a small number of target nodules. As a result, the therapeutic effect of DEM1-TACE in this study is currently limited to cases where the tumor size is relatively small and only a few nodules are targeted. Therefore, future studies using larger, multicenter cohorts are needed to validate the findings of our study.

## Conclusions

DEM1-TACE showed a high treatment ability for HCC. Especially, homogeneous contrast medium retention after DEM1-TACE had a significantly high ORR compared with the absence of homogeneous contrast medium retention. Therefore, the evaluation of homogeneous contrast medium retention after DEB-TACE by IVR-CT is one of the useful predictors of treatment success. In addition, the dilution rate is a critical factor when considering DEB-TACE using DC Bead M1^TM^. Maintaining an appropriate high dilution rate is also important for achieving good treatment effects in DEM1-TACE.
